# Growth inhibition of *Candida* species by *Wickerhamomyces anomalus* mycocin and a lactone compound of *Aureobasidium pullulans*

**DOI:** 10.1186/1472-6882-14-439

**Published:** 2014-11-08

**Authors:** Sun-Tee Tay, Su-Lin Lim, Hui-Wee Tan

**Affiliations:** Department of Medical Microbiology, Faculty of Medicine, University of Malaya, 50603 Kuala Lumpur, Malaysia

**Keywords:** *Aureobasidium pullulans*, *Candida*, 5-hydroxy-2-decenoic acid lactone, Glucanase, Mycocin, *Wickerhamomyces anomalus*

## Abstract

**Background:**

The increasing resistance of *Candida* yeasts towards antifungal compounds and the limited choice of therapeutic drugs have spurred great interest amongst the scientific community to search for alternative anti-*Candida* compounds. Mycocins and fungal metabolites have been reported to have the potential for treatment of fungal infections. In this study, the growth inhibition of *Candida* species by a mycocin produced by *Wickerhamomyces anomalus* and a lactone compound from *Aureobasidium pullulans* were investigated.

**Methods:**

Mycocin was purified from the culture supernatant of an environmental isolate of *W. anomalus* using Sephadex G-75 gel filtration column chromatography. The mycocin preparation was subjected to SDS-PAGE analysis followed by MALDI TOF/TOF mass spectrometry analysis. The thermal and temperature stability of the mycocin were determined. The glucanase activity of the mycocin was investigated by substrate staining of the mycocin with 4-methyl-umbelliferyl-ß-D-glucoside (MUG). Gas chromatography mass spectrometry (GCMS) analysis was used to identify anti-*Candida* metabolite in the culture supernatant of an environmental isolate of *Aureobasidium pullulans*. The inhibitory effects of the anti-*Candida* compound against planktonic and biofilm cultures of various *Candida* species were determined using broth microdilution and biofilm quantitation methods.

**Results:**

A mycocin active against *Candida mesorugosa* but not *C. albicans*, *C. parapsilosis* and *C. krusei* was isolated from the culture supernatant of *W. anomalus* in this study. The mycocin, identified as exo-ß-1,3 glucanase by MALDI TOF/TOF mass spectrometry, was stable at pH 3–6 and temperature ranging from 4-37°C. The glucanase activity of the mycocin was confirmed by substrate staining with MUG. 5-hydroxy-2-decenoic acid lactone (HDCL) was identified from the culture supernatant of *A. pullulans*. Using a commercial source of HDCL, the planktonic and biofilm MICs of HDCL against various *Candida* species were determined in this study.

**Conclusions:**

*W. anomalus* mycocin demonstrated a narrow spectrum of activity targeting only against *C. mesorugosa*, while HDCL demonstrated a broad spectrum of inhibitory action against multiple *Candida* species. The growth inhibition of *W. anomalus* mycocin and the lactone compound from *A. pullulans* against *Candida* yeasts should be further explored for therapeutic potentials against candidiasis.

## Background

Mycocinogenic or “killer” yeasts secrete proteinaceous mycocins (also known as killer toxins) that are lethal to other yeast strains but to which the producing yeasts are immune [1, 2]. Since the first discovery of killer toxin-secreting strains of *Saccharomyces cerevisiae* [3], yeast killer phenomenon has been documented in many different yeast species and genera from the environment, including *Wickerhamomyces anomalus* (previously known as *Pichia anomala* and *Hansenula anomala*) and *Aureobasidium pullulans* [4–9]. Both yeasts have been reported to have many applications in food fermentation, biocontrol and production of therapeutic molecules [7, 10]. The antifungal activity of *W. anomalus* has been associated with glucanase-induced lysis [7], while the production of many metabolites, enzymes and antibiotics by *A. pullulans* has been important for its biocontrol activity [10].

*Candida* is a medically important yeast pathogen which causes mucocutaneous and life-threatening systemic infections in susceptible individuals. Recent investigation based on molecular approach suggests that the genus is not monophyletic, and the relationships among various *Candida* species are not clearly resolved [11]. Amongst the *Candida* species, *Candida albicans* is the most predominant cause of candidiasis; however, the incidence of infections caused by non-albicans *Candida* species such as *C. rugosa* species complex has also increased [12, 13].

Candidiasis is usually associated with biofilm formation on the indwelling medical devices. Biofilms cells are embedded within an extracellular matrix and are difficult to treat as the cells are significantly more resistant to antimicrobial agents [14, 15]. Several mycocins have been reported to have the potential for treatment against fungal infections including candidiasis [16–18]. Additionally, microbial metabolites such as ethanol, isoamyl alcohol, 2-phenylethanol, 1-dodecanol, E-nerolidol, glycolipid biosurfactant and signalling molecules secreted in the yeast culture filtrates have been known to affect the growth of *C. albicans* biofilm [19, 20]. For instance, farnesol is a quorum-sensing molecule which inhibits pseudohyphae transition and biofilm formation in *C. albicans* [21, 22].

Due to the increasing resistance of *Candida* towards antifungal compounds and the limited choice of therapeutic drugs, searching for new antifungal compound is necessary. *W. anomalus* and *A. pullulans* with mycocinogenic activity have been isolated from fermented food and the natural environment in our study recently [8, 9]. Herein, we describe our investigation on the growth inhibition of *Candida* species by *W. anomalus* mycocin and a lactone compound from *A. pullulans*.

## Methods

### Yeasts and assessment of mycocin activity

*W. anomalus* strain tp2-15 and *A. pullulans* strain L7-10 isolated from fermented tapioca and a plant leaf, respectively, from two previous studies were used in this study [8, 9]. Identification of yeast strains was performed by sequence analysis of the yeast internal transcribed spacer (ITS) regions. Analysis using BLAST (Basic Local Alignment Search Tool) at NCBI database revealed 100% sequence similarities of the strain tp2-15 with *W. anomalus* strain MUCL 51259 from Belgium (585/585 nucleotides, Genbank accession no. FN394001) [23]. The strain tp2-15 differed in 1 nucleotide (gap) compared to the type strain of *W. anomalus* (AY046221). The strain L7-10 showed 100% sequence similarity with that of *A. pullulans* isolate BK6 from Thailand (574/574 nucleotides, Genbank accession no. AY225164) [24]. Queries at ISHAM ITS database (http://its.mycologylab.org/) show that the sequence of strain tp2-15 was 100% similarity with several *W. anomalus* reference sequences (MITS501, 502, 492, 490, 511, 520, 513–516) in the database, while the sequence of strain L7-10 was most identical (99.827% similarity, 1 gap) with the reference sequence of a *A. pullulans* strain (MITS374). ITS sequence of the strain L7-10 showed 99% similarity to the neotype strain of *A. pullulans* var. *pullulans* (FJ150906), 99% similarity to the type strain of *A. pullulans* var. *namibae* (FJ150875), 99% similarity to the type strain of *A. pullulans* var. *melanogenum* (FJ150886) and 98% similarity to the type strain of *A. pullulans* var. *subglaciale* (FJ150895). The closest match among the type strains (6 substitutions) was observed with the neotype strain of *A. pullulans* var. *pullulans* and the neotype strain of *A. pullulans* var. *namibae*.

The sensitivity strains used for assessment of mycocinogenic activity in this study were *C. albicans* ATCC 90028, *C. parapsilosis* ATCC22019 and *C. krusei* ATCC6258. Additionally, due to the unavailability of reference strains, clinical cultures of *C. dubliniensis* (designated as strain 2879) [25] and a member of *C. rugosa* species complex (designated as 3209, Genbank accession no. HM641831) [26] were also used. According to the recent reassessment of the *C. rugosa* species complex, the strain 3209 has been re-identified as *C. mesorugosa* [13].

A well diffusion assay was used for assessment of the mycocin activity, as described previously [8, 9]. Briefly, overnight cultures of sensitive strain were suspended in sterile distilled water to obtain about 10^5^ cells/ml (80% transmittance at 530 nm followed by 10X dilution). The yeast suspension was spread using a sterile swab on a YEPD-MB agar plate. Yeasts were then point-inoculated in duplicate on the agar plate. The plate was incubated at 30°C for 72 h and inspected daily for the appearance of inhibition zone surrounding the inoculated area as an indication of mycocinogenic activity.

As the inhibitory effect of *W. anomalus* was observed only against *C. rugosa* complex as demonstrated in the previous study [8], the same strain (3209) was used as a sensitive strain for subsequent study. For testing of anti-*Candida* metabolite from *A. pullulans*, *C. albicans* reference strain SC5314 was used for assessment of *A. pullulans* metabolites in the study. Additionally, a total of 92 clinical isolates from various *Candida* species were used to investigate the inhibitory effects using broth microdilution and biofilm quantitation methods. The isolates included *C. glabrata* (n =26), *C. krusei* (n =17), *C. albicans* (n =15), *C. parapsilosis* (n =8), *C. tropicalis* (n =10), *C. orthopsilosis* (n =8), *C. rugosa* species complex (n =5), *C. dubliniensis* (n =1), *Meyerozyma guilliermondii* (n =1) and *C. metapsilosis* (n =1). These isolates had been identified using API 20C AUX system (bioMerieux, Marcy, l’Etoile, France) or sequence analysis of their ITS gene regions prior to susceptibility testing.

### Isolation and purification of *W. anomalus*mycocin

Strain tp2-15 was cultivated in YEPD broth (1000 ml) buffered with 50 mM sodium phosphate citric acid (pH 4.5) at 30°C and 120 rpm in a gyratory shaker (Stuart, UK) for 3 days. The culture supernatant was filtered through a 0.22 μm filter system (Sartorius, Germany) and added with ice-cold ethanol (Fisher Scientific, USA) to a final concentration of 70% (v/v). The mixture was then incubated at -20°C for 1 h and centrifuged at 12,000 rpm at 4°C for 45 min. The pellet was resuspended in 10 ml of 50 mM sodium phosphate citric acid buffer (pH 4.5) incorporated with 20% glycerol and filtered. For determination of pH stability, mycocin samples were prepared in 50 mM sodium phosphate citric acid buffered at different pHs (ranging from 3 to 6). The mycocin activity was assessed by a well diffusion assay as described above except that the samples were pipetted into wells of approximately 10 mm in diameter in the YEPD-MB agar plate. For thermal stability study, mycocin samples were buffered at pH 4.5 and incubated at different temperatures: 4, 25, 30, 37, 50, 70 and 100°C for 1 h before the mycocin activity was assessed.

For purification of the mycocin, Sephadex G-75 gel slurry (product code: 17-0050-01, lab packs, GE healthcare, USA) in 50 mM sodium phosphate citric acid buffer (pH 4.5) was packed into a column of 1.8 cm (diameter) × 100 cm (length) to 80 cm in length and equilibrated with the same buffer at a rate of 1 ml/min. Seven milliliters of the above mycocin preparation were then loaded onto a Sephadex G-75 gel-filtration column. Elution was performed using the same buffer at a flow rate of 1 ml/min at 4°C. Fractions of 2 ml were collected and filtered through 0.2 μm pore size filters (Sartorius, Germany) for mycocin testing. The presence of protein in the fractions was determined by measuring the UV absorbance (at 280 nm) using a Nanophotometer™ (Implen GmbH, Germany). Fractions with mycocin activity were pooled and concentrated using a 10 kDa-cut off ultrafilter (Vivaspin 20, Sartorius, Germany). The mycocin activity was determined using a microplate assay as described by Hodgson *et al*. [16]. Briefly, 100 μl mycocin preparation was mixed with equal volume of *C. mesorugosa* cell suspension pre-adjusted to 10^5^ cells/ml in RPMI 1640 medium (Sigma-Aldrich, USA) in a microtiter well and incubated at 30°C for 24 h. Yeast growth was determined by measuring OD_450_ using a microplate reader (Glomax, Promega, USA). Mycocin activity was expressed as the percentage growth inhibition of the sensitive strain compared to a mycocin-free control (i.e. 50 mM sodium phosphate citric acid buffer, pH 4.5).

SDS-PAGE was performed according to Laemmli [27] using a concentrated sample from the active fractions in a mini-PROTEAN® Tetra Cell electrophoresis system (Biorad, USA) A 4% (w/v) acrylamide was used in the stacking gel and 10% (w/v) acrylamide was used in the resolving gel. After electrophoresis, the protein was stained with Coomassie brilliant blue R-250 (Sigma-Aldrich, USA) and the molecular mass was determined by comparison with known marker proteins (Fermentas, Lithuania). The protein fragments in the SDS-PAGE gel was identified by MALDI TOF/TOF mass spectrometry (Proteomics International, Australia) using a 5800 Proteomics Analyzer (AB Sciex, USA).

Discontinuous Native PAGE was performed to detect glucanase activity from the active fractions as described by Nebrada *et al*. [28]. After electrophoresis, the native gel was incubated with 0.2% 4-methyl-umbelliferyl-ß-D-glucoside (MUG) (Sigma-Aldrich: M-3633, USA) in 50 mM acetate buffer (pH 5.0) at 30°C for 45 min. Protein with glucanase activity will be visualized as a fluorescence band on a UV transilluminator (Syngene, UK).

### Isolation and identification of a lactone compound from *A. pullulans*

Strain L7-10 was cultured in a broth containing 0.67% (w/v) yeast nitrogen base medium supplemented with 2% (w/v) glucose at 30°C with agitation (120 rpm) for seven days. The culture broth was then separated from the biomass by centrifugation at 4000 rpm for 30 min and filtered through a 0.22 μm filter membrane (Sartorius Stedim Biotech GmbH, Germany). A total of 12 liters of culture filtrate was extracted three times with ethyl acetate before fractionating using a 40 × 100 mm Prep Nova-Pak HR C18 column (Waters Co., Milford, MA, USA) in a Prep-HPLC system. Twelve fractions (A to L) were collected and each fraction was adjusted to 1 mg/ml prior to testing using a biofilm quantitation method [9].

The identification of compound in the active fractions was performed using Shimadzu GC-17A gas chromatography, GCMS-QP5050A mass spectrometer and a capillary column Zebron ZB-1 (30 m X 0.25 mm X 0.25 μm diameter). GC-MS was performed using splitless injection with the following setting: injector at 250°C, column at 50°C, heating ramp of 5°C/min, final temperature of 300°C, and MS/FID detector at 280°C. Helium was used as a carrier gas at 1.4 ml/min. The GC-MS electron ionization system was set at 70 eV. Identification of active compound was made by comparison with the database from WILEY 229 library of mass-spectra, National Institute of Standards and Technology (NIST) 107 and NIST 21.

Due to the limited availability of the lactone compound for testing, 5-hydroxy-2-decenoic acid lactone (abbreviated as HDCL, CAS no: 54814-64-1) was obtained from Penta International Corp., Livingston, USA. A stock solution of HDCL was prepared at 450 mg/ml in DMSO (AMRESCO, Cleveland, Ohio, USA). For determination of the susceptibility of 92 isolates of various *Candida* species, final concentrations of HDCL ranging from 8 to 4096 μg/ml were prepared in RPMI 1640 broth medium with L-glutamine, without bicarbonate (Sigma Aldrich, USA), and buffered with MOPS medium (pH 7.0). The MICs of the isolates were determined by broth microdilution method as in accordance to CLSI method (2008).

The biofilm MIC of the isolates was determined as described by Melo *et al*. [29]. Briefly, an overnight yeast culture was suspended in RPMI 1640 broth medium supplemented with L-glutamine and buffered with MOPS (Sigma, USA) and adjusted to 10^7^ cells/ml (OD_530nm_ = 0.38) prior to use. One hundred microliters of the yeast suspension was then transferred to each well of a flat-bottomed, 96-well polystyrene microtiter plate. After incubation under shaking condition (75 rpm) at 37°C for 1.5 h, unattached cells were removed by washing twice with 150 μl phosphate-buffered saline (PBS). Fresh RPMI 1640 medium (100 μl) was then added to the microtiter well to allow proliferation of the biofilm for 24 h. The biofilm cells were washed once with PBS before exposure to 100 μl of HDCL solutions (final concentration ranging from 8 to 4096 μg/ml). The metabolic activity of biofilms after 48 hours of exposure to HDCL was assessed using XTT reduction assays as described by Jin *et al*. [30]. Briefly, the biofilm in each well was washed twice with 200 μl PBS before adding with a solution containing 2,3-bis(2-methoxy-4-nitro-5-sulfophenyl)-5-(phenylamino)carbonyl)-2*H*-tetrazolium hydroxide (XTT) with menadione. The absorbance of the resultant solution (100 μl) after incubation for 1 h in the dark was measured using a spectrophotometer at 492 nm. The biofilm MIC of each *Candida* isolate was determined on the basis of a 50% reduction in metabolic activity compared with the metabolic activity of the biofilm without exposure to HDCL. The experiments were performed in duplicate.

## Results

### Isolation and identification of *W. anomalus*mycocin

**Figure 1:**
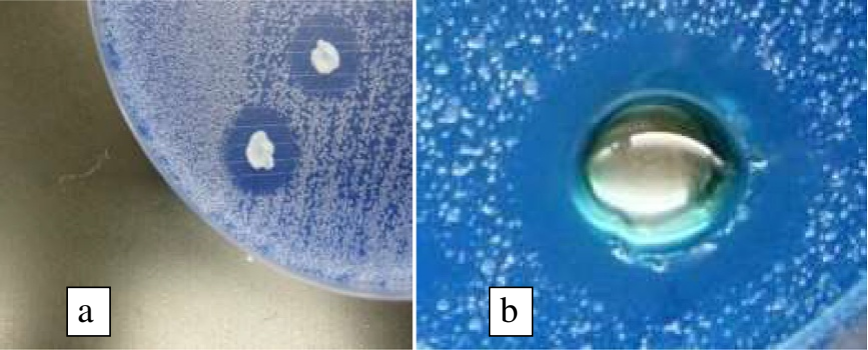
**Killer activity by (a) cultures of** ***W. anomalus*** **killer yeast and (b) an ethanol-precipitated mycocin sample, against** ***C. mesorugosa*** **on a YEPD-MB agar plate.**

**Figure 2:**
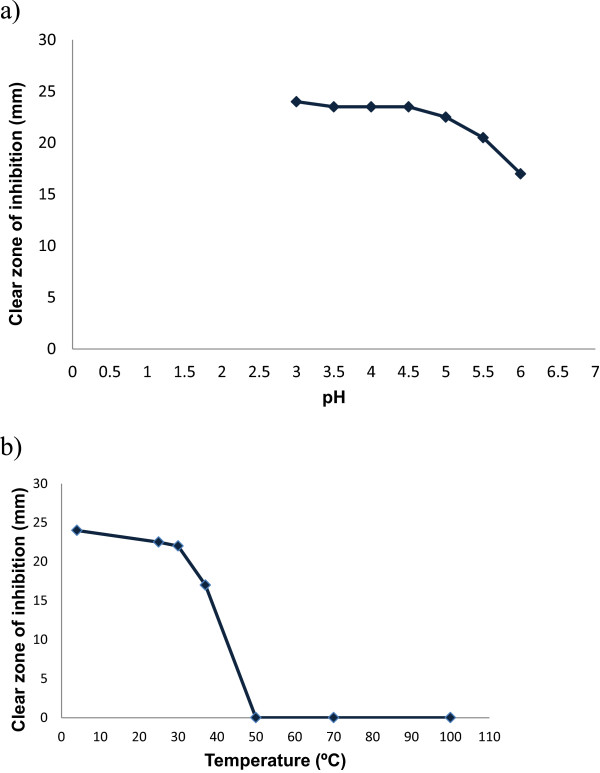
**Determination of the effect of (a) pH and (b) temperature on the activity of** ***W. anomalus*** **mycocin.**

**Figure 3:**
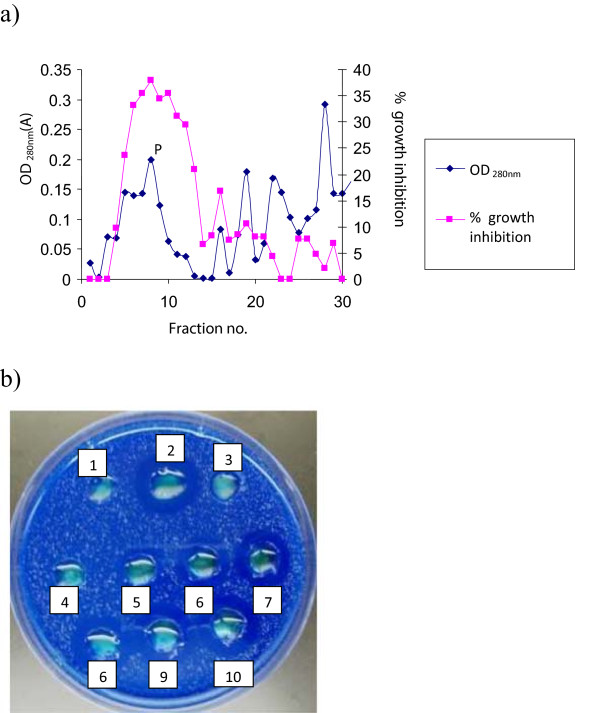
**Determination of the mycocin activity of fractions eluted from Sephadex gel filtration column against** ***C. mesorugosa*** **by microplate assay (a): the highest growth inhibition against** ***C. mesorugosa*** **was shown in fraction 8 which corresponded with a protein peak (P); and well diffusion assay (b): 1, void sample; 2, ethanol-precipitated mycocin sample; 3 to 10, 1 to 8 eluted fractions.**

Figure 1 shows the inhibitory effects of *W. anomalus* culture and the ethanol-precipitated mycocin preparation against *C. mesorugosa* (strain 3209) using well diffusion assay on a YEPD-MB agar plate. Clear zone of inhibition (about 2–3 mm from the edge to the colony/well) was observed for both *W. anomalus* culture and the ethanol-precipitated mycocin preparation after 2 days of incubation. Figure 2 shows that the mycocin was active at pH 3–6 when incubated at 30°C. The mycocin activity was detected when incubated at 4, 25, 30 and 37°C, but no activity was detected beyond 50°C. A total of 30 fractions were eluted from the Sephadex G-75 gel column for determination of mycocin activity. Fraction 8 demonstrated the highest growth inhibition against the yeast by microplate assay (Figure 3a) and well diffusion assay (Figure 3b).

**Figure 4:**
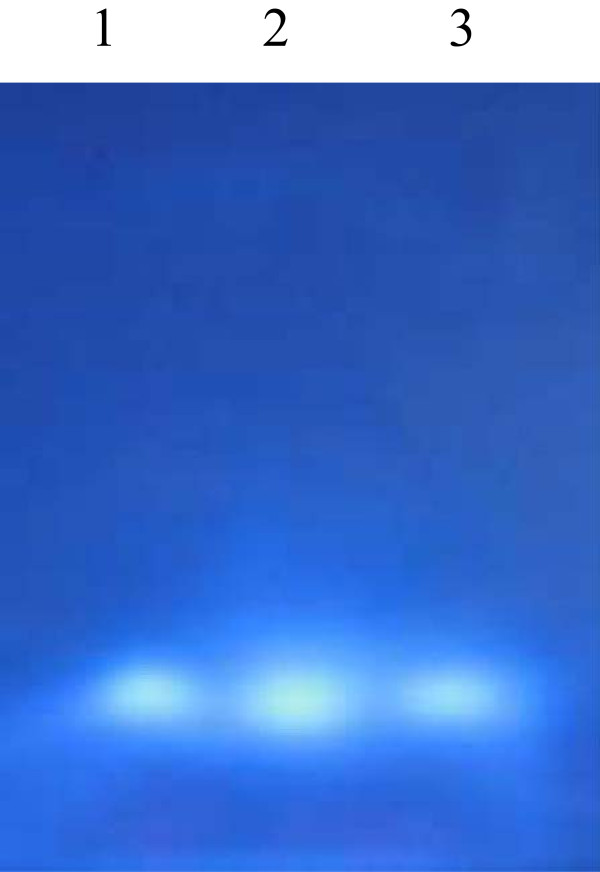
**A fluorescent activity band was observed in the ethanol-precipitated culture supernatant of three batches of** ***W. anomalus*** **, indicating the presence of ß-1,3-glucanase activity (Lane 1 to 3).** ß-1,3-glucanase activity was detected by overlaying the native gel with 0.2% (wt/vol) MUG in acetate buffer (pH 5) and incubating for 45 min at 30°C before UV illumination.

SDS-PAGE analysis of a concentrated active fraction revealed four major protein fragments (data not shown). Mass spectrometry analysis demonstrated the identification of two fragments (45 kDa and 50 kDa) as exo-ß-1,3-glucanase. Both fragments demonstrated the closest match to that of the glucanase derived from a marine yeast, *P. anomala* YF07b (A0MPR7) which inhibited the growth of *Metschnikowia bicuspidata*, a pathogenic yeast causing milky disease in crab [31]. A peptide sequence of GDYWDYQND KIR was detected from the fragment with the molecular weight of 45 kDa, while three matching peptides (GDYWDYQND KIR, LNDFWQQGYHNLR and WLNGVGR) were detected from the fragment with the molecular weight of 50 kDa. MUG staining of native gel with the mycocin samples showed the presence of fluorescent activity bands, indicating the association of glucanase activity with the *W. anomalus* mycocin (Figure 4).

### Isolation and purification of lactone compound from *A. pullulans*

**Figure 5:**
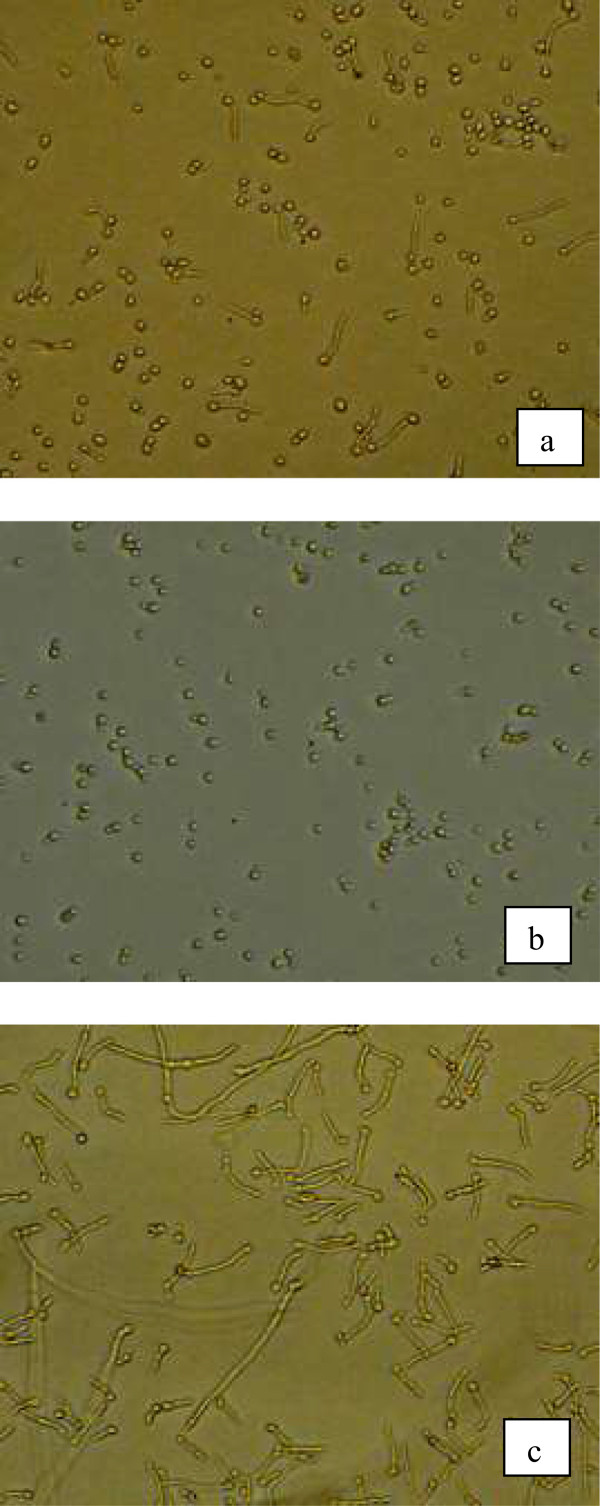
**The effects of (a) fraction J (b) fraction K derived from culture supernatant of** ***A. pullulans*** **on the biofilm development of** ***C. albicans*** **reference strain SC5314 (yeast cells were co-incubated with crude extract and allowed to proliferate for 24 h) (c) negative control (normal growth medium was used instead of crude extract).**

**Figure 6:**
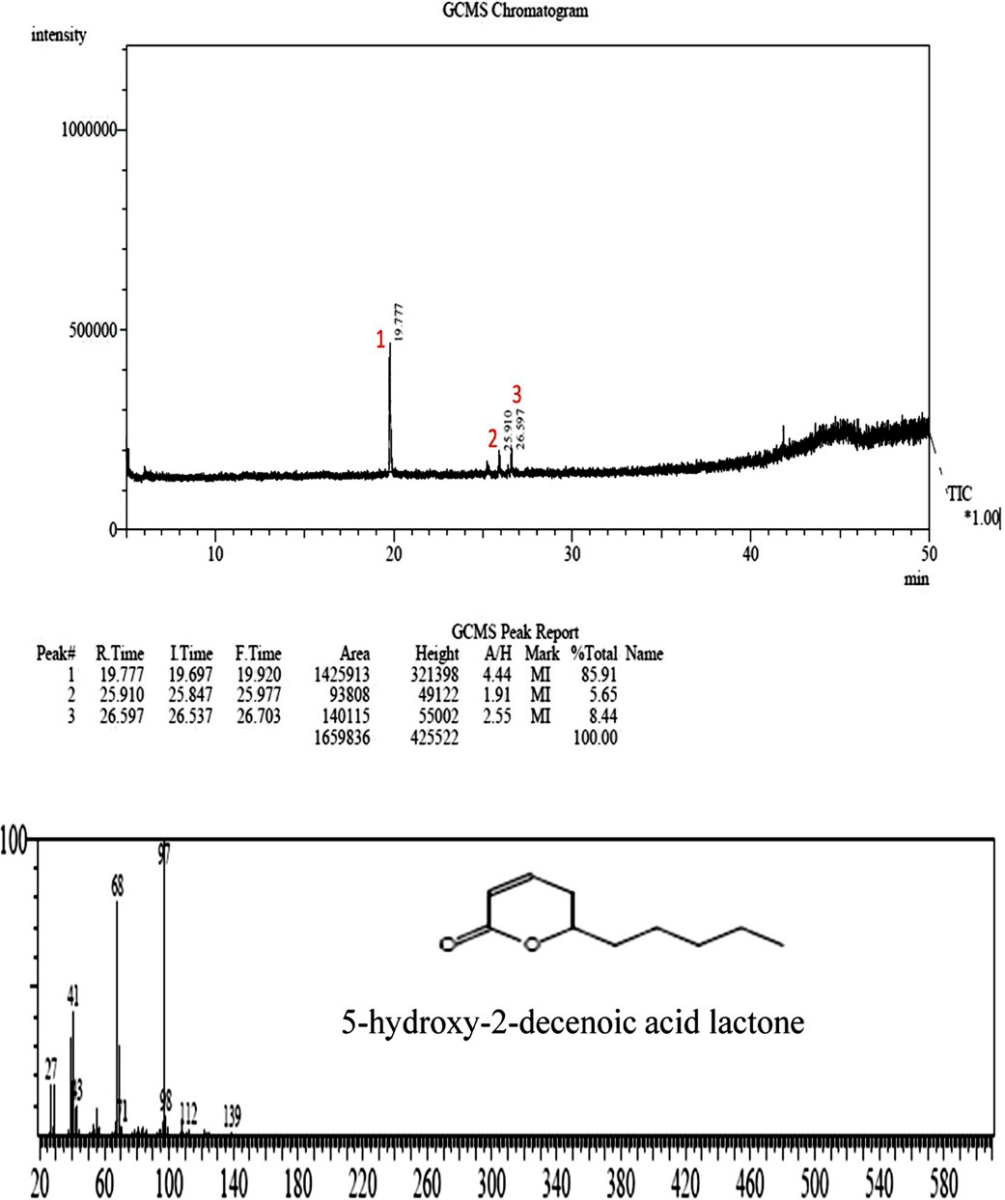
**GC-MS analysis of the chemical structure and mass spectrum of the major compounds present in the fraction K of** ***A. pullulans*** **culture extract.**

Only two fractions (J and K) exhibited inhibitory effect against biofilm cultures of *C. albicans*. Microscopic analysis showed inhibition of hyphal extension by fractions J and K, whereas extensive hyphal filaments were observed for the negative control (untreated cells) after 72 h of incubation (Figure 5). GC-MS analysis revealed the presence of 5-hydroxy-2-decenoic acid lactone (95% match, Figure 6) in the fraction K. The lactone compound is characterized by a closed ring containing two or more carbon atoms and a single oxygen atom, with a ketone group (=O) in one of the carbons adjacent to the other oxygen.

Table 1 shows the susceptibility profiles of *Candida* isolates to the commercial source of 5-hydroxy-2-decenoic acid lactone (abbreviated as HDCL). The planktonic MICs range, MIC_50_ and MIC_90_ of HDCL against the isolates were 64 to 256 μg/ml, 256 μg/ml and 256 μg/ml, respectively. Compared to other isolates, *Candida parapsilosis* isolates showed the lowest MIC_50_ values (64 μg/ml). The planktonic MIC_50_ and MIC_90_ of non-albicans *Candida* species including *Candida krusei*, *C. glabrata*, *C. parapsilosis*, *C. tropicalis*, *C. orthopsilosis* and *C. rugosa* species complex were 256 μg/ml. The biofilm MIC range, MIC_50_ and MIC_90_ of the *Candida* yeasts were 64 to 1024 μg/ml, 256 μg/ml and 512 μg/ml, respectively (Table 1). Compared to other species, *Candida krusei*, *C. parapsilosis* and *C. orthopsilosis* isolates exhibited the lowest MIC_50_ values (128 μg/ml). The overall biofilm MIC_50_ and MIC_90_ of non-albicans *Candida* species were both 256 μg/ml.

**Table 1:** ***In vitro*** **susceptibilities of** ***Candida*** **isolates to a standard preparation of HDCL**

***Candida***spp.	Planktonic MIC (μg/ml)			Biofilm MIC (μg/ml)		
	MIC range	MIC _50_	MIC _90_	MIC range	MIC _50_	MIC _90_
*Candida albicans* ( n = 15)	128 - 256	256	256	128 - 512	256	256
Nonalbicans *Candida* species (n = 77)	64 - 256	256	256	64 - 1024	256	256
*Candida glabrata* (n = 26)	64 - 256	256	256	64 - 512^a^	256^a^	512^a^
*C. krusei* (n = 17)	128 - 256	128	256	64 - 256	128	256
*C. parapsilosis* (n = 8)	64 - 256	64	256	128 - 1024^b^	128^b^	1024^b^
*C. tropicalis* (n = 10)	64 - 256	128	256	256 - 1024	256	512
*C. orthopsilosis* (n = 8)	128 - 256	256	256	128 - 256^c^	128^c^	256^c^
*C. rugosa* species complex (n = 5)	128 - 256	128	256	128 - 512	512	512
*C. dubliniensis* (n =1)	128	ND	ND	256	ND	ND
*M. guelliermondii* (n =1)	128	ND	ND	128	ND	ND
*C. metapsilosis* (n =1)	256	ND	ND	128	ND	ND
**All isolates (n = 92)**	**64 - 256**	**256**	**256**	**64 - 1024**	**256**	**512**

Footnote: Planktonic MIC: The lowest concentration of HDCL required to inhibit yeast growth after incubation at 37°C for 48 h.

Biofilm MIC: The lowest concentration of HDCL required to cause 50% reduction in biofilm metabolic activity after incubation at 37°C for 48 h.

MIC_50_ and MIC_90_ values were only calculated for those species with 5 or more isolates.

ND: Not determined.

^a, b, c^: Five *Candida glabrata* and two each of *C. parapsilosis* and *C. orthopsilosis* isolates were excluded from the study due to poor biofilm formation (OD_490nm_ < 0.1).

## Discussion

Research in mycocinogenic yeasts has improved our understanding on the eukaryotic cell biology, host-virus interaction and the mechanisms of eukaryotic secretion pathway [2, 3, 5, 6]. *W. anomalus* (previously known as *P. anomala*) producing anti-*Candida* toxins have been reported in several studies [32–34]. In this study, the protein isolated from *W. anomalus* exhibited basic characteristics of mycocin with pH 3–6 and temperature ranging from 4 to 37°C. Additionally, the mycocin was identified as exo-ß-1,3 glucanase by mass spectrometry analysis and its activity was confirmed by MUG staining.

Glucanase is produced by yeasts to facilitate morphogenesis and recycling of cell wall components [35, 36]. Table 2 summarizes the features of mycocins derived from four *P. anomala* strains (WC65, NCYC 432, 434, YF07b) which had been purified by various chromatographic methods [34, 37, 38]. All mycocins were active at low pH. The mycocin for WC65 strain had the highest molecular weight (83.3 kDa) compared to others [37, 38]. The mycocin isolated from this study shared similar characteristics (pH, temperature and molecular weight) as those reported for NCYC 432, 434, YF07b strains [37, 38]. However, unlike other mycocins from *P. anomala* NCYC 434 and WC 65 which demonstrated inhibition against the growth of *C. albicans*, the mycocin investigated in this study was not inhibitory to *C. albicans*, instead, it was found to be only active against *C. mesorugosa*.

**Table 2:** **Source and features of** ***W*** **.** ***anomalus*** **mycocins**

***W***. ***anomalus***strain	WC 65 [34]	NCYC 434 [37]	NCYC 432 [38]	YF07b [31]	tp2-15 (isolated in this study)
Source	Culture collection	Culture collection	Culture collection	Marine- derived	Fermented tapioca
pH stability	2 to 5	3 to 5.5	3 to 5.5	3 to 5	3 to 6
Temperature stability (°C)	Not determined	Up to 37	Up to 37	20-90	4 to 37
Molecular weight (kDa)	83.3	49	47	47	45, 50
pI	5.0	3.7	3.4/3.7	-	Not determined
Amino acid sequence analysis	Not determined	Homology with exo-ß-1,3 glucanase (accession no. AJ222862)	Homology with exo-ß-1,3 glucanase (accession no. AJ222862)	Exo-ß-1,3 glucanase (A0MPR7)	Homology with exo-ß-1,3 glucanase (A0MPR7)
Inhibitory activity	*C. albicans*, *Cyberlindnera bimundalis*, *Cyberlindnera jadinii*, *Kluyveromyces lactis*, *Saccharomyces cerevisiae*, *Saccharomycodes ludwigii*	*C. albicans*, *Torulaspora delbrueckii*, *Kluyveromyces marxianus*, *Saccharomyces cerevisiae*	*C. albicans*, *C. tropicalis*, *C. pseudotropicalis*, *C. glabrata*, *C. parapsilosis*, *C. krusei*	*Metschnikowia bicuspidata*	*C. mesorugosa*

The different killer pattern of *W. anomalus* mycocin could be attributed to the specificity of glucanases which have selective preference for different types of glycosidic linkages and glucan receptors of the sensitive yeast strains [6, 39–41]. Several investigators have proposed the use of mycocin for clarification of the taxonomy of closely related organisms [33, 42]. Recently, *C. rugosa* has been differentiated to separate clade from other pathogenic *Candida* species including *C. albicans*, *C. tropicalis*, *C. parapsilosis*, based on multigene analyses [11]. Hence, the finding on the killer pattern of *W. anomalus* mycocin may reflect the different levels of taxonomic organization of this organism when compared to *C. albicans*.

Mycocin has been proposed as a novel agent for treating fungal infections [5, 16–18, 42]. The fact that the target molecule for mycocin (glucan as for *W. anomalus* mycocin in this study) is absent in mammalian cells, suggests its potential application against fungal infections on the skin and mucosal membranes, as most mycocins are not suitable for oral or intravenous use, due to their sensitivity to proteinase degradation, antigenicity, and activity within a narrow pH range [42]. However, development of anti-idiotypic antibodies which share the active site of the mycocin and possess antifungal activity is possible [5, 18].

In this study, a lactone compound (5-hydroxy-2-decenoic acid lactone) inhibiting various *Candida* species was isolated from an ethyl-acetate extract of the culture supernatant of *A. pullulans*. Lactone is an analogue of 6-pentyl-2H-pyran-2-one (synonym: massoialactone), which has been reported as a byproduct of *Trichoderma* [43]. Some specific isolates of *Aureobasidium* also produced large amounts of delactonised massoialactone as an extracellular ester [44]. *In vitro* biological activity of massoialactone-related compounds against phytopathogens and human pathogens including *C. albicans* has been reported [43, 45]. Several lactone compounds have been reported to be effective at preventing biofilm formation of bacteria or fungi. For instance, plant sesquiterpene lactones are inhibitory to the biofilm growth of *P. aeruginosa* [46] and 3-oxo-C_12_ homoserine lactone (3-oxo-C_12_-HSL) is inhibitory to *C. albicans* filamentation by blocking the yeast-to-hyphal switch [47]. Unlike amphotericin, azoles and caspofungin which target at the fungal cell wall, the antimicrobial mechanism responsible for massoialactone activity against *C. albicans* is probably through the inhibition of the fungal respiratory system [44]. However, data is limited as there has been no detail study on the use of the lactone compound against *Candida* yeasts.

HDCL demonstrated a broad spectrum of inhibitory action against multiple *Candida* species in this study. However, a high concentration of HDCL (at least 64 μg/ml) was required to inhibit both planktonic and biofilm cultures of *Candida* yeasts. In our previous study, the amphotericin and fluconazole MIC_50_ against a culture collection of Malaysian clinical isolates of *C. albicans* and non-albicans *Candida* species were 0.25 and 2 μg/ml, respectively [48]. An increased resistance of *Candida* biofilm cultures to amphotericin (more than 32 folds) and fluconazole (≥128 μg/ml) as compared to their planktonic counterparts was also reported in that study. In this study, there was no obvious difference in the HDCL MICs of both planktonic and biofilm cultures of *Candida* yeasts (Table 1).

## Conclusion

The growth inhibition of *W. anomalus* mycocin and 5-hydroxy-2-decenoic acid lactone from *A. pullulans* against *Candida* yeasts should be further explored for biotechnological applications and therapeutic potentials. Pathogenic fungi such as *Aspergillus* and dermatophytes may be included for future testing to broaden the spectrum of mycocin and the lactone compound. As little is known about yeast killing phenomena and mechanisms of actions, proteome profiling and gene expression studies of *Candid*a in response to these molecules will be required.
